# Universal Clinical DDH Screening Complemented with Targeted Ultrasound Is Effective in Finland

**DOI:** 10.2106/JBJS.24.00313

**Published:** 2025-02-20

**Authors:** Emma-Sofia Luoto, Jenni Jalkanen, Ilari Kuitunen, Reijo Sund, Yrjänä Nietosvaara

**Affiliations:** 1 Department of Pediatric Surgery, Kuopio University Hospital, University of Eastern Finland, Kuopio, Finland; 2 Department of Pediatrics, Kuopio University Hospital, Kuopio, Finland; 3 Kuopio Musculoskeletal Research Unit, Institute of Clinical Medicine, University of Eastern Finland, Kuopio, Finland; 4 Department of Pediatric Orthopedics and Traumatology, New Children’s Hospital, Helsinki University Hospital, Helsinki, Finland

## Abstract

**Background:**

The late diagnosis rate of developmental dysplasia of the hip (DDH) with universal ultrasound screening is 0.2 per 1,000 children according to a recent meta-analysis, which is the same as in Japan where selective ultrasound screening is used. We hypothesized that Finland’s current program of universal clinical screening complemented with targeted ultrasound is noninferior to universal and selective ultrasound screening programs.

**Methods:**

For this retrospective cohort study, we collected the number of children <15 years of age who were diagnosed with DDH (International Classification of Diseases, Tenth Revision [ICD-10] codes Q65.0-Q65.6 and Ninth Revision [ICD-9] code 7543) as their primary diagnosis after ≥3 visits to a physician. These data were obtained from the Finnish Care Register for Health Care, which collects the ICD-10 and ICD-9 codes from every medical appointment. We calculated the annual incidence of DDH diagnoses per 1,000 newborns between 2002 and 2021. Late diagnosis of DDH was defined as a finding of DDH in children aged 6 months through <15 years at the initial diagnosis who had undergone treatment under anesthesia (closed reduction and casting or surgery). We also registered the geographic, age, and sex distributions of the DDH diagnoses.

**Results:**

During the 20-year study period, 1,103,269 babies were born (median per year, 57,214 babies; range per year, 45,346 to 60,694 babies). A total of 6,421 children had a diagnosis of DDH (mean per year, 321 children; range per year, 193 to 405 children), with a mean calculated incidence of 5.8 per 1,000 newborns (95% confidence interval [CI], 5.7 to 6.0). Altogether, 120 children aged 6 months through <15 years were treated for DDH, with little annual variation (median, 6.5 children; range, 2 to 9 children). The mean national incidence of late-diagnosed cases was 0.11 per 1,000 newborns (95% CI, 0.09 to 0.13).

**Conclusions:**

Finland’s current DDH screening program, which includes universal clinical screening with targeted ultrasound, is noninferior when compared with other screening programs.

**Level of Evidence:**

Prognostic Level III. See Instructions for Authors for a complete description of levels of evidence.

Developmental dysplasia of the hip (DDH) encompasses a wide spectrum of conditions that range from minor acetabular dysplasia with a stable hip to irreducible hip dislocation^1^. Early diagnosis of hip instability is imperative because late diagnosis is associated with an increased likelihood of surgery, more intrusive procedures, and worse clinical outcomes^2^. However, which screening program should be applied to detect DDH is still a topic for debate. A universal ultrasound screening program is standard practice in some European countries, such as Germany and Austria, whereas a clinical or selective ultrasound screening policy exists in Scandinavia, the United Kingdom, and the United States^3,4^.

The current screening policy for DDH in Finland has been in place for >30 years. It entails a clinical examination of every newborn by a hospital pediatrician before discharge from the maternity hospital. Newborns showing positive Ortolani and/or Barlow signs (indicating an unstable hip) or equivocal findings are referred to a pediatric orthopaedic outpatient clinic for reexamination within 2 weeks. If spontaneous stabilization occurs by the time of reexamination, no treatment is initiated; however, a repeat examination and ultrasonography of the hip are conducted at 6 weeks after the first outpatient clinic visit.

If a newborn’s hips have not stabilized by 2 weeks of age, we start a 6-week treatment with a von Rosen splint, except in the Tampere University Hospital Catchment Area (TAUH), where a Pavlik harness is used. General practitioners at cost-free child welfare clinics screen for DDH at 4 to 6 weeks, 4 months, 8 months, and 18 months (Fig. 1).

**Fig. 1:**
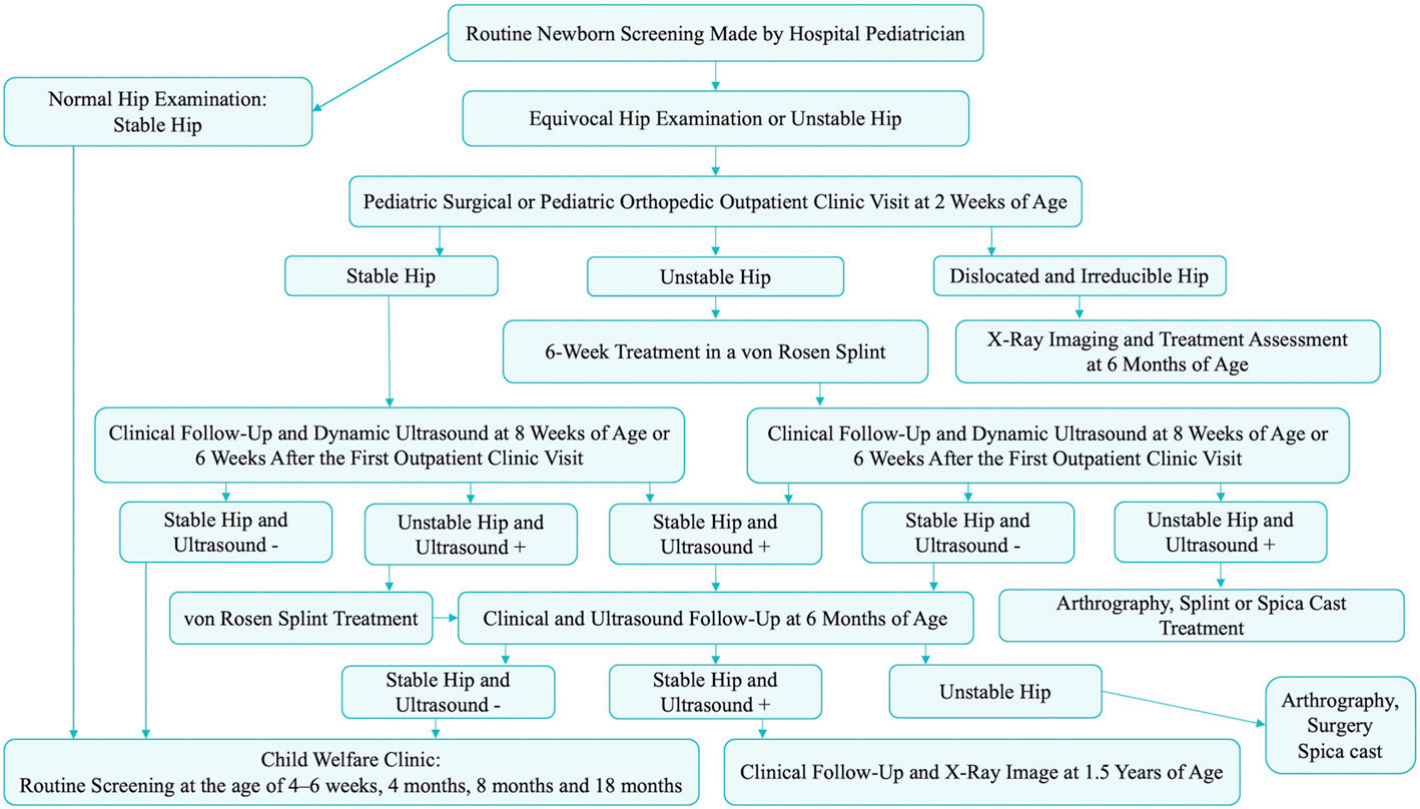
Current screening program and treatment policy for DDH in Finland. Ultrasound − represents a normal hip ultrasound finding, Ultrasound + represents an abnormal hip ultrasound finding, Ortolani + and Barlow + indicate a positive sign in the maneuver in question, and Ortolani − and Barlow − indicate a negative sign in the maneuver.

There are no recent studies on late-diagnosed DDH in Finland. A study by Heikkilä et al. reported an incidence of 0.76 per 1,000 children with universal clinical screening in Uusimaa County, in southern Finland, between 1966 and 1975^5^. In addition, according to a recent meta-analysis, universal ultrasound screening does not seem to be more effective: the incidence of late-diagnosed DDH with universal ultrasound screening was approximately 0.2 per 1,000 children ^6^. In our study, we aimed to establish a more up-to-date incidence of late-diagnosed DDH in Finland. Our hypothesis was that Finland’s current screening program of universal clinical screening complemented with targeted ultrasound is noninferior compared with the efficacy of other reported screening programs.

## Materials and Methods

Ethical approval was obtained from the Research Ethics Committee of the Northern Savo Hospital District (58/2021).

### Data Sources

The Care Register for Health Care (HILMO), managed by the Finnish Institute for Health and Welfare, contains comprehensive health-care data. It includes information on service providers, procedures, discharge diagnoses according to the ICD-10 and ICD-9 (International Classification of Diseases, Tenth and Ninth Revisions), and patient details, such as municipality of residence, personal identity code, age, and sex.

### Patient Identification Process

For this retrospective cohort study, we collected the number of children younger than 15 years of age who were diagnosed with DDH (ICD-10 codes Q65.0-Q65.6 and ICD-9 code 7543) as their primary diagnosis after ≥3 outpatient clinic visits. An inclusion criterion was implemented to exclude children with neonatal hip instability who had spontaneous recovery: follow-up appointments are discontinued once the hips are noted as stable in 2 consecutive clinical examinations and appear normal in ultrasound images.

Only children who were born between January 1, 2002, and December 31, 2021, were included in the study. We registered the patients’ geographic distributions using the University Hospital catchment areas and assessed the age and sex distribution of DDH diagnoses. All of the municipalities in Finland, and, therefore, the patients, were part of 1 of the 5 ERVA (University Hospital Catchment Area) areas. Patients with coexisting neuromuscular conditions, congenital abnormalities, or other syndromes that were likely to predict teratological DDH were excluded from the study. Only 1 entry per personal identity code was included in the search. Lastly, to compute the incidence rates, we collected the annual numbers of live births in Finland between January 1, 2002, and December 31, 2021, from Statistics Finland^7^.

### Late Diagnosis

To identify late-diagnosed DDH cases, we collected the number of children aged 6 months through <15 years who were diagnosed with DDH and who subsequently received surgery (Nordic Classification of Surgical Procedures [NCSP] codes NFE10, NFE15, NFH10, NFH20, NFL20, NFL22, NFK30, and NEL10) or were treated with a spica cast (NCSP codes TNX33, TNX34, and TNF34) for DDH. This identification method was based on the current clinical practice in Finland: a hip arthrogram followed by treatment under anesthesia (closed reduction and casting or surgery) was performed after the age of 6 months, while splint treatment was applied before reaching that age. Children who had experienced failed splinting or harness treatment before 6 months of age were excluded.

### Statistical Analysis

We estimated the incidence rate of DDH per 1,000 newborns by dividing the total number of DDH cases in a particular birth-year cohort by the total number of live births in that same year, and then multiplying the result by 1,000. The incidence rate of late diagnosis was calculated accordingly using the late-diagnosed DDH cases. Confidence intervals (CIs) for the incidences were calculated by assuming a Poisson distribution for the number of events. The cumulative incidences of DDH from birth to <15 years of age and late-diagnosed DDH from 6 months to <15 years of age were calculated. Both linear and spline models were fitted to assess the trends of yearly DDH and late-diagnosed DDH. The spline model did not differ significantly from the linear model in either case. Therefore, examining the slope of the fitted linear model was considered sufficient for testing linearity.

To avoid underestimation due to incomplete follow-up, we used competing-risks survival analysis to account for right-censoring, ensuring valid cumulative incidence estimates. For the trend analysis, we included current-year late DDH diagnoses from earlier birth cohorts. Assuming consistent practices over time and supported by cumulative incidence data and full follow-up years, we derived reliable estimates for both incidences and trends. Statistical analysis was performed with SPSS Statistics (version 27.0 for Mac; IBM).

## Results

### Incidence of DDH

**Fig. 2:**
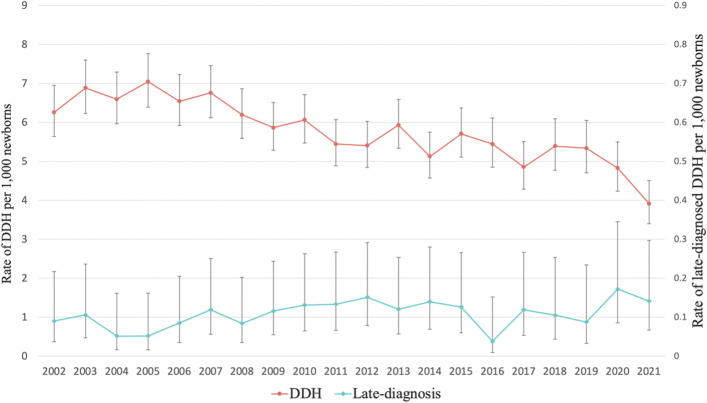
The annual incidences of DDH and late-diagnosed DDH per 1,000 newborns in Finland between 2002 and 2021. The whiskers indicate 95% CIs.

During the 20-year study period, a total of 1,103,269 babies were born (median per year, 57,214 babies; range per year, 45,346 to 60,694 babies)^7^. A total of 6,421 children were diagnosed with DDH (mean per year, 321 children; range per year, 193 to 405 children) during the study period, giving a mean calculated incidence rate of 5.82 per 1,000 newborns (95% CI, 5.68 to 5.96). The slope of the fitted line was −0.119 per year per 1,000 live births, and a significant change was found in the yearly incidence of DDH during the study period (p < 0.0001) (Fig. 2).

### Age and Sex Distribution of DDH

**Fig. 3:**
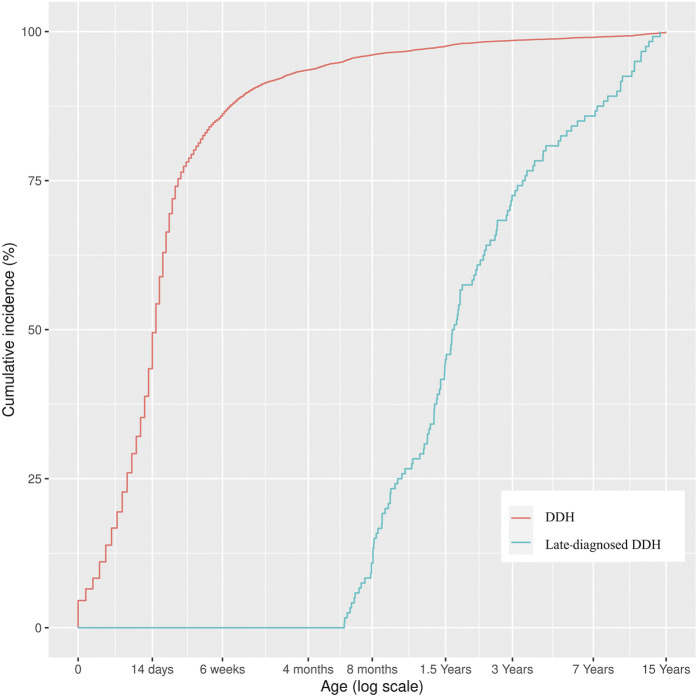
The cumulative incidence of DDH diagnosed from birth to age <15 years and the cumulative incidence of late-diagnosed DDH in Finland from 6 months to <15 years.

The highest incidence of receiving a DDH diagnosis occurred in the group aged 0 to 14 days (3.14 per 1,000 newborns; 95% CI, 3.04 to 3.25), followed by the group aged 15 to 90 days (2.42 per 1,000 newborns; 95% CI, 2.34 to 2.52) and the group aged 91 to 180 days (0.10 per 1,000 newborns; 95% CI, 0.08 to 0.11). The stated age groups accounted for 97.0% of all diagnosed cases of DDH. Thereafter, the incidence of receiving a DDH diagnosis decreased gradually from 0.05 per 1,000 newborns (95% CI, 0.04 to 0.06) in the age group ranging from 180 to 365 days to its lowest number at 0.03 per 1,000 newborns (95% CI, 0.02 to 0.04) in the age group ranging from 6 to <15 years (Fig. 3). The incidence of DDH for girls was 4.40 per 1,000 newborns, (95% CI, 4.28 to 4.53), whereas the incidence for boys was 1.42 per 1,000 newborns (95% CI, 1.35 to 1.49). Lastly, the female-to-male incidence rate ratio (IRR) of DDH was 4.40 (95% CI, 4.27 to 4.53).

### Geographic Distribution of DDH

**Fig. 4:**
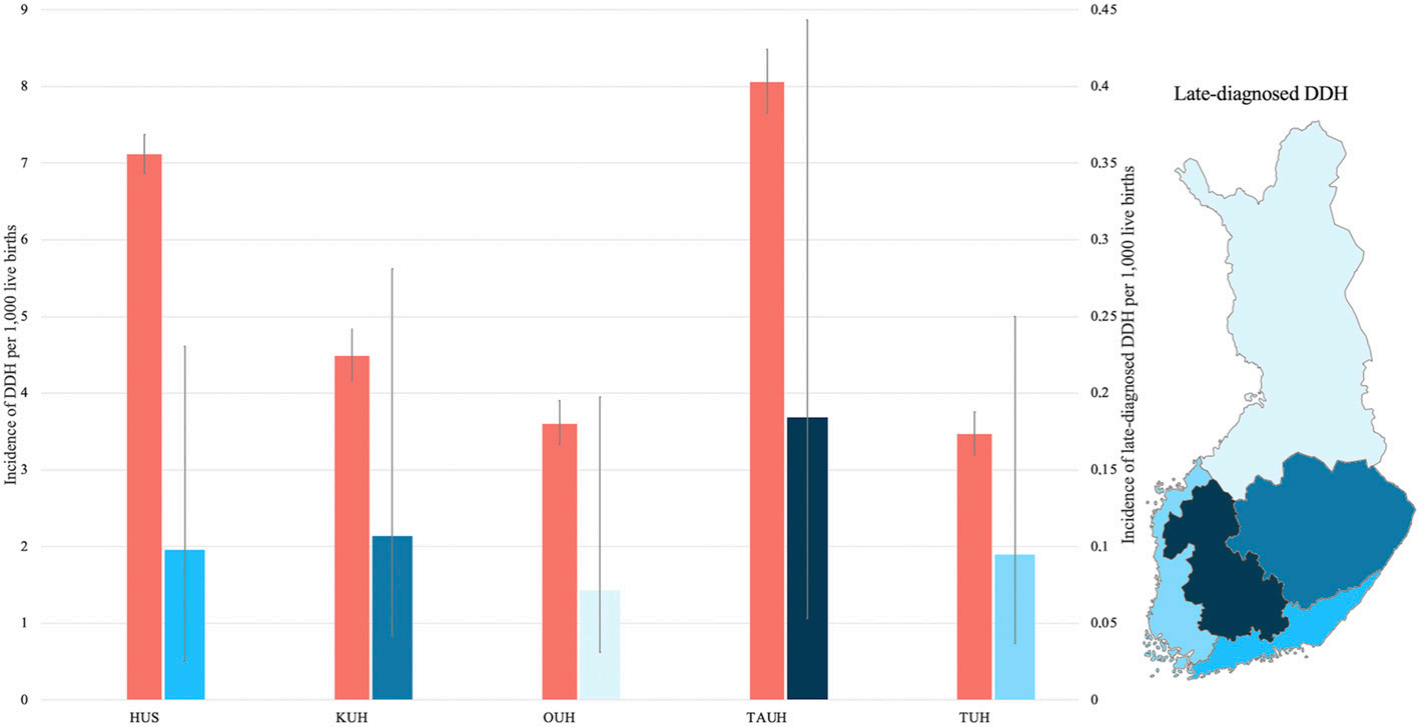
The incidences of DDH and late-diagnosed DDH per 1,000 live births between 2002 to 2021 listed by hospital catchment area, with 95% confidence intervals. HUS = Helsinki University Hospital, KUH = Kuopio University Hospital, OUH = Oulu University Hospital, TAUH = Tampere University Hospital, and TUH = Turku University Hospital.

The incidence of receiving a DDH diagnosis was highest in the TAUH catchment area (8.06 per 1,000 newborns; 95% CI, 7.65 to 8.48). The lowest incidence was found in the Turku University Hospital (TUH) catchment area (3.46 per 1,000 newborns, 95% CI, 3.19 to 3.75). Children born in the TAUH catchment area were 4.60 times (95% CI, 4.10 to 5.10) more likely to be diagnosed with DDH compared with those born in the TUH catchment area, and were 2.24 times (95% CI, 1.80 to 2.70) more likely to be diagnosed than were children born across the entire study area (Fig. 4, Table I).

**TABLE I:** Geographic Distribution of DDH and Late-Diagnosed DDH in Finland According to Hospital Catchment Area

Hospital Catchment Area	No. of Live Births	No. of DDH Cases	Incidence of DDH per 1,000 Newborns*	No. of Late-Diagnosed DDH Cases	Incidence of Late-Diagnosed DDH per 1,000 Newborns*
Helsinki University Hospital (HUS)	438,224	3,118	7.12 (6.87-7.37)	43	0.10 (0.07-0.13)
Kuopio University Hospital (KUH)	149,788	672	4.49 (4.16-4.84)	16	0.11 (0.07-0.17)
Oulu University Hospital (OUH)	167,651	604	3.60 (3.33-3.90)	12	0.07 (0.04-0.13)
Tampere University Hospital (TAUH)	179,120	1,443	8.06 (7.65-8.48)	33	0.18 (0.13-0.26)
Turku University Hospital (TUH)	168,486	584	3.46 (3.19-3.75)	16	0.09 (0.06-0.16)
Total	1,103,269	6,421	5.82 (5.68-5.96)	120	0.11 (0.09-0.13)

* The values are given as the value with the 95% CI in parentheses.

### Incidence of Late-Diagnosed DDH

Altogether, 120 children (median, 6.5 children; range, 2 to 9 per year; 0.11 per 1,000 newborns; 95% CI, 0.09 to 0.13 per 1,000 newborns) between the ages of 6 months and <15 years were treated for DDH during the 20-year study period, with little annual variation (0.04 to 0.17 per 1,000 newborns). The slope of the fitted line was 0.003 per year per 1,000 live births, and the change in the annual incidence of late-diagnosed DDH during the study period did not reach significance (p = 0.058) (Fig. 2).

### Age and Geographic Distribution of Late-Diagnosed DDH

The majority of late-diagnosed DDH cases were identified before the age of 2 years (Fig. 3). The distribution of late-diagnosed DDH was concentrated in the TAUH catchment area (0.18 per 1,000 newborns; 95% CI, 0.13 to 0.26), whereas the lowest incidence rate was found in the Oulu University Hospital (OUH) catchment area (0.07 per 1,000 newborns; 95% CI, 0.04 to 0.13). Children born in the TAUH catchment area were 2.57 times (95% CI, 1.33 to 4.98) more likely to have a late diagnosis of DDH than were children born in the OUH catchment area, and were 1.69 times (95% CI, 1.15 to 2.49) more likely to have a late diagnosis than children who were born in the study area as a whole (Fig. 4, Table I).

## Discussion

This was a nationwide study reporting the trends in receiving a DDH diagnoses and the incidence of late-diagnosed DDH during a 20-year period. Our main finding was the low rate of late-diagnosed DDH cases with universal clinical screening complemented with targeted ultrasound when compared with the results of other reported screening policies.

The incidences of DDH and late-diagnosed DDH are markedly impacted by the screening method, the timing of said screening, and the precise definition of DDH^8,9^. Nationwide studies on the incidences of DDH and late-diagnosed DDH are often limited. This underscores the need to adopt a cautious approach when comparing incidence rates. Nonetheless, the incidence of DDH with universal ultrasound screening was 18.2 per 1,000 newborns in a population-based study that was conducted in Pomerania, a region in northeast Germany^10^. This contrasts with our findings for Finland, where the nationwide incidence was 5.8 per 1,000 newborns with universal clinical screening complemented with targeted ultrasound. Lastly, in Japan, the incidence of DDH with universal risk-based selective screening was only 0.76 per 1,000 newborns^11^.

In Germany and Austria, incidence rates for late-diagnosed DDH requiring treatment under anesthesia were 0.16 and 0.26 per 1,000 newborns, respectively, with both countries using universal ultrasound screening^12,13^. The incidence rate of DDH that was diagnosed with selective ultrasound screening after the age of 6 months was 0.15 per 1,000 newborns in Japan^11^. In England, an incidence rate of 1.28 per 1,000 newborns was found for DDH that was diagnosed with selective ultrasound screening after 12 months of age^14^. A study conducted in Sweden, where a screening program similar to the one in Finland is used, reported an incidence rate of 0.07 per 1,000 newborns for children who were diagnosed with DDH after the age of 6 months, but the study only included children who were <4.5 years of age^15^.

A universal clinical DDH screening program has been implemented in Japan and Finland. In Japan, routine assessment for DDH involves newborn home visits and health checkups within 4 months that are conducted by public health nurses and midwives^16^. Conversely, in Finland, newborns are examined by a hospital pediatrician, and 4 additional checkups are conducted by general practitioners. While Japan employs routine ultrasonography for children with known risk factors, Finland only uses ultrasonography for children with clinically detected or suspected hip instability. These differing screening procedures may contribute to the observed differences in the reported incidence rates of DDH in Japan and Finland.

There was a significant decreasing trend (p < 0.0001) in the annual incidence of DDH in Finland between 2002 and 2021. Conversely, the incidence of late-diagnosed DDH showed a slight upward trend during the same period, although this trend did not reach significance (p = 0.085). This discrepancy may suggest a potential decline in the effectiveness of the current screening policy; however, caution should be used when comparing the incidence trend of late-diagnosed DDH with the incidence trend of DDH. The incidence of DDH may be less reliable than that of late-diagnosed DDH due to variability in the use of ICD codes for confirmed and suspected cases when compared with the more standardized use of surgical procedure codes. The accuracy of diagnostic coding for DDH has likely improved since the early 2000s, when the current screening policy for DDH in Finland was introduced, reducing the occurrence of false positives in subsequent years. There has also been a 40% decrease in the number of birth centers in Finland from 2002 to 2021. Thus, smaller health-care providers may have utilized the DDH diagnostic code more liberally, while larger ones may have been more likely to use it only for true hip instability. Unfortunately, these cases cannot be differentiated in our register data.

There was a twofold increase in the number of children between the ages of 0 and <15 years who migrated to Finland between 2002 and 2021^17^, which may also have influenced the number of late diagnoses because these children had not been screened for hip instability at birth in Finland. Lastly, the incidence trend of late-diagnosed DDH is more likely to be influenced by minimal changes because the number of late-diagnosed DDH cases is considerably smaller compared with the number of early-diagnosed DDH cases.

We found some regional differences in the incidence of DDH. These discrepancies may be a result of screening practices, especially when considering the more consistent incidences of late-diagnosed DDH cases across the University Hospital catchment areas. The differing incidence of late-diagnosed DDH may be a result of a poorer standard of clinical hip examination, differences in follow-up protocols, or genetic differences between populations, considering that the reported incidence of DDH varies with ethnicity and geographic location^18^.

Universal ultrasound screening is often advocated, with the aim of reducing late-diagnosed DDH cases. Previous randomized controlled trials and meta-analyses have, however, shown that the rate of late diagnosis is not zero, even when there is 100% compliance with universal ultrasound screening^19^. Moreover, the evidence from randomized controlled trials does not support the notion that universal ultrasound screening is effective in reducing the incidence of late-diagnosed DDH, and the most favorable outcomes that have been reported in nationwide cohorts are associated with programs that depend on neonatal clinical screening that is conducted by more experienced or specifically trained staff^9,15,20-22^. Finally, our current strategy of universal clinical screening complemented with targeted ultrasound seems to be successful and is associated with one of the lowest nationwide incidence rates of late-diagnosed DDH globally. Thus, the need for universal ultrasound screening should be critically assessed because implementation of universal screening in Finland would not provide much value but would notably increase costs^6,23,24^ (Table II).

**TABLE II:** Comparison of Screening Methods for DDH

Screening Method	Factors Leading to Outcome	Notable Characteristics	Additional Considerations
Clinical screening plus selective ultrasound	Physician’s clinical experience and expertise	False negatives due to reliance on physical examination False positives due to subjective interpretation Cost-effective	Limited sensitivity and specificity May lead to missed or delayed diagnoses
Universal ultrasound screening	Availability of ultrasound equipment Operator’s training and skill level	False negatives due to operator error or technical limitations False positives due to overinterpretation or misidentification More accurate in diagnosing DDH	Higher initial cost May detect cases missed by clinical examination May lead to unnecessary treatment or anxiety in false- positive cases

This study’s main strength lies in its population-based design, allowing for precise nationwide incidence estimates and the observation of temporal and regional differences^25^. However, one limitation was that we assessed the total number of DDH cases relative to live births rather than calculating the ratio of total DDH cases to the population’s total at-risk time for the disease. In addition, a control group would have given the conclusion more weight. We also acknowledge that there were potential issues related to information bias stemming from the diverse application of diagnostic criteria. Additionally, there may have been measurement bias due to potential inaccuracies and inconsistencies of diagnostic coding^26^. Additional studies should explore reasons for late diagnoses and the regional differences that were identified in this study.

In conclusion, the current national clinical screening program that is complemented with targeted ultrasound in children with detected or suspected hip instability in the maternal hospitals or in child welfare centers is noninferior to the reported efficacy of selective and universal ultrasound protocols. In Finland, the number of hip instability cases diagnosed after 6 months of age is low, and, in most of these cases, the diagnosis is made before the age of 2 years.
